# New Antimicrobial Bioactivity against Multidrug-Resistant Gram-Positive Bacteria of Kinase Inhibitor IMD0354

**DOI:** 10.3390/antibiotics9100665

**Published:** 2020-10-01

**Authors:** Iliana E Escobar, Alexis White, Wooseong Kim, Eleftherios Mylonakis

**Affiliations:** 1 Infectious Diseases Division, Department of Medicine, Warren Alpert Medical School of Brown University, Rhode Island Hospital, Providence, RI 02903, USA; iliana_escobar@brown.edu (I.E.E.); alexis_white@brown.edu (A.W.); 2 College of Pharmacy, Graduate School of Pharmaceutical Sciences, Ewha Womans University, Seoul 03760, Korea

**Keywords:** high-throughput screening, vancomycin-resistant *Staphylococcus aureus*, vancomycin-resistant enterococci, IMD0354

## Abstract

Multidrug-resistant pathogens pose a serious threat to human health. For decades, the antibiotic vancomycin has been a potent option when treating Gram-positive multidrug-resistant infections. Nonetheless, in recent decades, we have begun to see an increase in vancomycin-resistant bacteria. Here, we show that the nuclear factor-kappa B (NF-κB) inhibitor *N*-[3,5-Bis(trifluoromethyl)phenyl]-5-chloro-2-hydroxybenzamide (IMD0354) was identified as a positive hit through a *Caenorhabditis elegans*–methicillin-resistant *Staphylococcus aureus* (MRSA) infection screen. IMD0354 was a potent bacteriostatic drug capable of working at a minimal inhibitory concentration (MIC) as low as 0.06 µg/mL against various vancomycin-resistant strains. Interestingly, IMD0354 showed no hemolytic activity at concentrations as high as 16 µg/mL and is minimally toxic to *C. elegans* in vivo with 90% survival up to 64 µg/mL. In addition, we demonstrated that IMD0354′s mechanism of action at high concentrations is membrane permeabilization. Lastly, we found that IMD0354 is able to inhibit vancomycin-resistant *Staphylococcus aureus* (VRSA) initial cell attachment and biofilm formation at sub-MIC levels and above. Our work highlights that the NF-κB inhibitor IMD0354 has promising potential as a lead compound and an antimicrobial therapeutic candidate capable of combating multidrug-resistant bacteria.

## 1. Introduction

Methicillin-resistant *Staphylococcus aureus* (MRSA) is a Gram-positive pathogen that can cause skin abscess, bloodstream infections, and pneumonia [1,2]. Infections associated with MRSA are among the leading hospital-acquired infections [3] They are associated with high mortality and increased hospital stays that result in a higher cost burden [3].

Vancomycin is a glycopeptide able to inhibit cell wall synthesis by binding to the ends of D-Ala-D-Ala moieties of un-crosslinked Lipid II molecules [4]. Vancomycin is an antibiotic effective at treating Gram-positive multidrug-resistant pathogens, including MRSA [4,5]. However, strains such as vancomycin-intermediate *Staphylococcus aureus* (VISA, minimal inhibitory concentration (MIC) = 4–8 µg/mL), vancomycin-resistant *Staphylococcus aureus* (VRSA, MIC ≥ 16 µg/mL), as well as vancomycin-resistant enterococci (VRE) have emerged [5,6,7,8,9].

During normal cell wall synthesis, penicillin-binding proteins (PBPs) are able to attach to terminal D-Ala-D-Ala moieties of un-crosslinked Lipid II and link them. Vancomycin is able to bind them and thus block PBPs’ attachment and crosslinking. This eventually leads to osmatic stress and bursting of the cell wall making vancomycin a potent bactericidal antibiotic [4].

Initially, it had been thought that resistance to vancomycin would be minimal given that it does not target enzymatic cell processes [4]. However, we now understand that vancomycin resistance is achieved by a group of genes encoding various enzymes and regulatory proteins that alter the original structure of Gram-positive bacterial walls [4,10,11]. These groups of genes are usually referred to as resistance cassettes. For vancomycin, the originally discovered cassette was named the “VanA”-type cassette which is composed of *vanHAX* cluster encoding enzymes and *vanR* and *vanS* genes that work as a two-component regulation system [12]. Numerous other resistance cassettes to vancomycin have been discovered and described, each of which includes VanA homologs [4]. These resistance cassettes encode genes that facilitate the conversion of D-Ala to D-Lac. In addition, other cassettes exist that help replace D-Ala with D-Ser [13].

Regardless of the change in the amino acid, the basic mechanism of resistance stays the same. By altering the original composition of Gram-positive Lipid II amino acid D-Ala-D-Ala vancomycin is no longer able to attach to the end of these glycopeptides and thus is unable to inhibit cell wall synthesis, creating bacteria mildly susceptible or resistant to vancomycin [4]. Given this threat, vancomycin resistance in Gram-positive bacteria poses a great risk to health care systems worldwide. As a last resort, antibiotics such as linezolid and daptomycin are clinically in use [14,15,16]. However, resistance to both drugs has become more prevalent throughout the decades [14,17,18]. Therefore, the development of new antibiotics to combat these drug-resistant bacteria is necessary and in dire need.

We screened ~82,000 small molecules to identify anti-infective agents that block *Caenorhabditis elegans* from a MRSA infection [19]. We identified several bioactive compounds, of which biological activities have been previously determined [20]. For example, the selective retinoic acid receptor γ (RARγ) agonist CD437 and CD1530 [19], the selective peroxisome proliferator-activated receptor γ (PPARγ)-agonist nTZDpa [21], the anti-parasite drug bithionol [22], and insulin-like growth factor receptor inhibitor PQ401 [23]. Each show promising antimicrobial potency against multidrug-resistant Gram-positive pathogens and their persister cells. Considering that many hit compounds are excluded for further investigation due to their in vivo inactivity and toxicity, bioactive compound hits in particular have a high potential to become lead compounds because their in vivo efficacy and in vivo toxicity have been previously proven in several animal models [21,22,23]. Therefore, we further investigated other bioactive compound hits.

In this study, we explore another bioactive compound hit *N*-[3,5-Bis(trifluoromethyl)phenyl]-5-chloro-2-hydroxybenzamide (IMD0354), previously described as an inhibitor of nuclear factor-kappa B (NF-κB) that works by directly blocking Iκκβ phosphorylation [24]. IMD0354 is known to have multiple biological activities. For instance, it has shown anti-cancer properties by directly inhibiting cell invasion and viability as well as acting as an adjuvant with other chemotherapy drugs without showing detectable toxicity [25,26,27]. In addition, it has also shown anti-inflammatory properties by blocking NF-κB and subsequent cytokine production [24,28]. Recently, it has been shown that IMD0354 potentiates colistin antimicrobial activity against colistin-resistant *Acinetobacter baumannii* [29]. However, the activity of this compound against Gram-positive bacteria and the activity of IMD0354 alone is not known to have antimicrobial potency. Here, we report, for the first time, that IMD0354 is notably potent against Gram-positive multidrug-resistant bacteria VRSA and VRE. We report that IMD0354 inhibits initial VRSA cell attachment and biofilm formation and is able to induce rapid membrane permeabilization at high concentrations of ≥ 4 µg/mL. Furthermore, we demonstrate that IMD0354′s antimicrobial activity is superior to its anti-cancer activity.

## 2. Results

### 2.1. IMD0354 Exhibits Anti-Staphylococcal Activity In Vitro & in a Whole Animal C. elegans Infection Model

**Figure 1:**
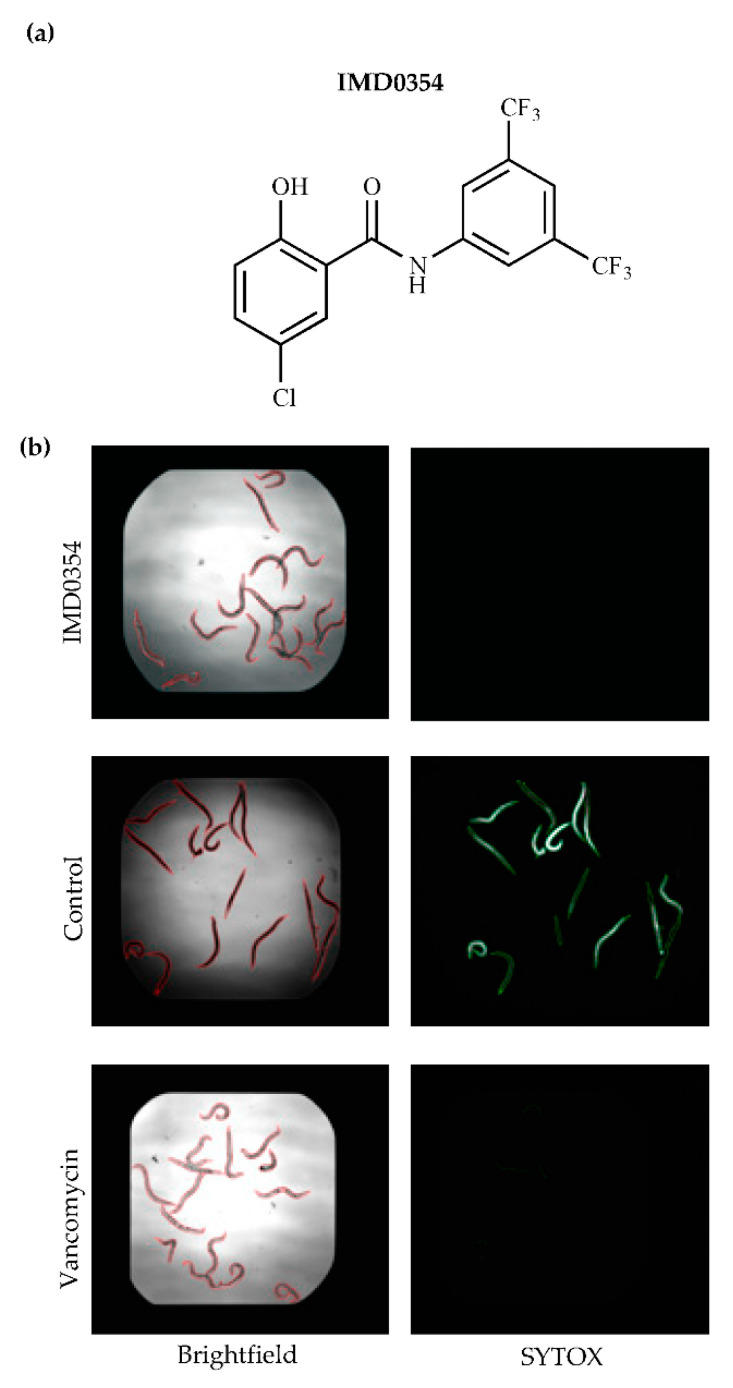
IMD0354 rescues *C. elegans* from MRSA infection. (**a**) Chemical structure of IMD0354. (**b**) Fifteen MRSA-infected *C. elegans* were treated with 7.14 µg/mL IMD0354, 0.1% dymethlyl sulfoxide (DMSO) (control) and 10 µg/mL vancomycin for 5 days. After staining dead worms with SYTOX Orange, brightfield (left) and fluorescence (right) images were obtained.

We identified the NF-κB inhibitor IMD0354 (Figure 1a) as a hit compound that impedes *C. elegans* from an MSRA infection (Figure 1b). This compound completely protected all 15 *C. elegans* worms from the MRSA infection at 7.14 µg/mL (Figure 1b). Upon further evaluation, we determined the protection mechanism by which IMD0354 functioned was direct antimicrobial activity against MRSA MW2 with a MIC of 0.06 µg/mL. Although IMD0354 has been known to have multiple bioactivities, such as anti-cancer, anti-inflammatory, and anti-viral activity [25,26,27], its direct antimicrobial activity has not been reported. Therefore, we focused on evaluating the potential of repurposing IMD0354 as an antimicrobial agent.

### 2.2. IMD0354 Shows Antimicrobial Activity against Multidrug-Resistant Gram-Positive Pathogens

First, we tested the antimicrobial activity of IMD0354 against ESKAPE pathogens that often cause nosocomial infections and acquire antibiotic resistance [30,31]. ESKAPE pathogens consist of two Gram-positive bacteria, *Enterococcus faecium* and *S. aureus*, and four Gram-negative bacteria, *Klebsiella pneumoniae, A. baumannii, Pseudomonas aeruginosa*, and *Enterobacter* species [30,31]. IMD0354 displayed antimicrobial potency against *E. faecium* E004 with a MIC of 0.125 µg/mL. In addition to *S. aureus* and *E. faecium*, IMD0354 exhibited significant antimicrobial activity against another Gram-positive bacterium, *Enterococcus faecalis* MMH 594, with a MIC of 0.25 µg/mL. Next, we assessed its antimicrobial potency against the four Gram-negative pathogens. A previous study with IMD0354 against *A. baumannii* showed that IMD0354 can work as a potent adjuvant, enhancing the antimicrobial potency of colistin, while having no or minimal antimicrobial activity on its own [29]. In our hand, IMD0354 showed low antimicrobial activity against *A. baumannii* strain ATCC 17978 with MIC of 16 µg/mL (Table 1). However, it was not potent against *P. aeruginosa* PA14, *K. pneumoniae* WGLW2, and *E. aerogenes* EAE 2625 (Table 1). Taken together these findings suggest that IMD0354 is effective against Gram-positive bacteria, while, in the absence of another agent such as colistin, it demonstrates limited antimicrobial activity against Gram-negative bacteria.

Next, we assessed the antimicrobial potency of IMD0354 against a panel of vancomycin-intermediate or -resistant strains, including the vancomycin-resistant strain VRS1 [32], 14 VISA clinical isolates acquired from the center for disease control and prevention (CDC) [33], and five VRE strains [33,34,35,36]. We found that the MIC ranged from 0.06 to 0.25 µg/mL for all vancomycin-resistant and vancomycin-intermediate *Staphylococcal* isolates (Table 2) and 0.25 µg/mL for all VRE strains (Table 3). Considering that the MICs of last-resort antibiotics, such as daptomycin and linezolid, are 1–2 µg/mL against VRSA and VRE [19,34], our results indicate that IMD0354 is a far more effective agent against these multidrug-resistant pathogens.

**Figure 2:**
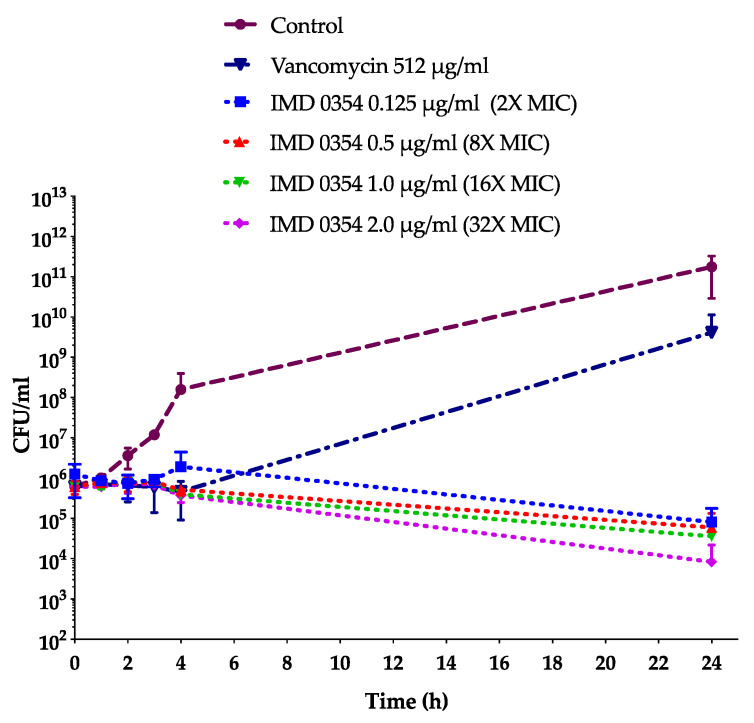
Time-killing curve shows IMD0354 is bacteriostatic against vancomycin-resistant strain VRS1. In total, 10^6^ CFU/mL of VRS1 overnight culture was treated with various concentrations of vancomycin and IMD0354. At times of 1, 2, 3, 4, and 24 h, samples we collected, serially diluted, and spot-plated in order to enumerate CFU/mL. Each sample was tested in triplicate. (*n* = 3, ± S.D.).

We then evaluated whether IMD0354 is bacteriostatic or bactericidal and found that IMD0354 is bacteriostatic to eight VISA strains (MBC/MIC ratio > 4) and bactericidal (MBC/MIC ratio ≤ 4) to six VISA strains (Table 2). Additionally, IMD0354 is bacteriostatic to a VRSA strain, VRS1, and all VRE strains tested (Table 2 and Table 3). These results were further confirmed by time-course killing kinetics of VRS1 cells treated with IMD0354 at 0.125 µg/mL (2× MIC), 0.5 µg/mL (8× MIC), 1 µg/mL (16× MIC), and 2 µg/mL (32× MIC) for up to 24 h (Figure 2). We found that IMD0354 was able to reduce colony forming units per mL (CFU/mL) by approximately 1 log in 24 h at a level of 8× to 16× MIC and up to 1.5 log at 32× MIC (Figure 2). However, vancomycin alone did not inhibit the bacterial growth of VRS1 [32] even at high concentrations such as 512 µg/mL (Figure 2).

### 2.3. Cytotoxicity of IMD0354 on Human Cells and C. elegans

**Figure 3:**
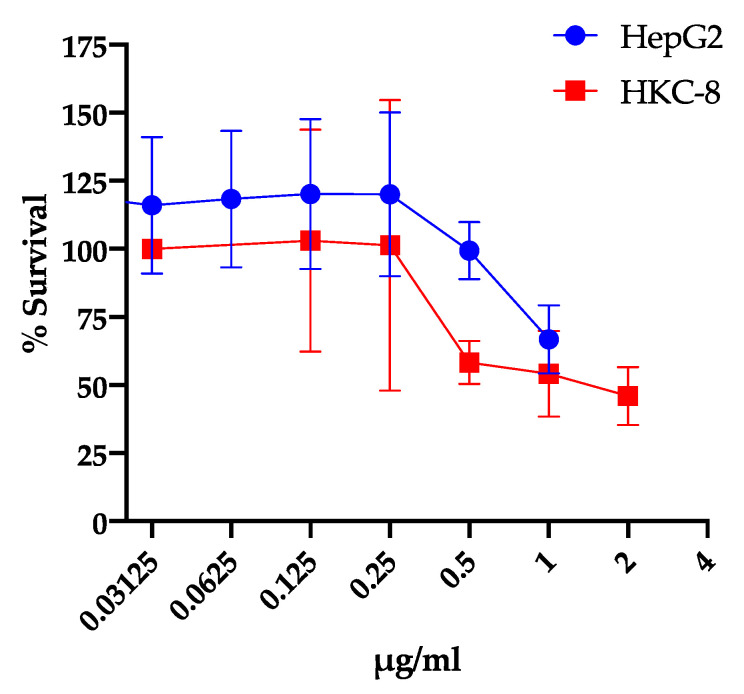
Dose toxicity studies show IMD0354 has cytotoxicity activity above MIC levels. Cytotoxicity testing of human liver cell line HepG2 and human kidney proximal tubular cell line HKC-8 at various concentrations of IMD0354. LC_50_ of IMD0354 is 1.1 µg/mL and is 0.94 µg/mL, respectively. Each sample was tested in triplicate. (*n* = 3, ± S.D.).

**Figure 4:**
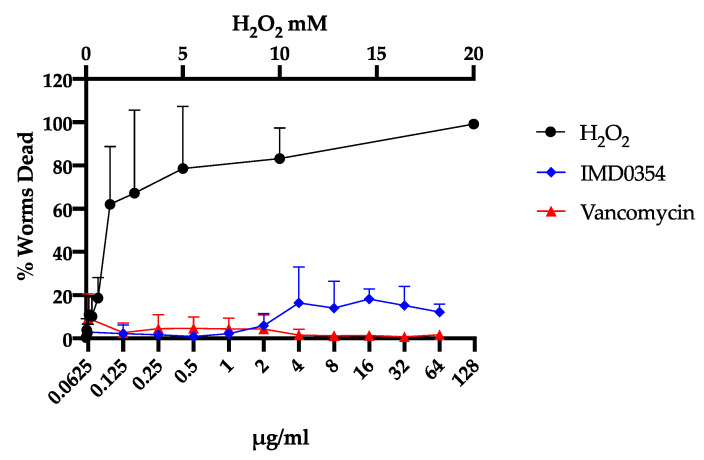
IMD0354 shows minimal toxicity toward *C. elegans*. Survival of *C. elegans* treated with various concentrations of IMD0354, normalized to *C. elegans* treated with DMSO. H_2_O_2_ was used as positive control. (*n* = 3, ± S.D). Bottom error bars are omitted for clarity.

**Figure 5:**
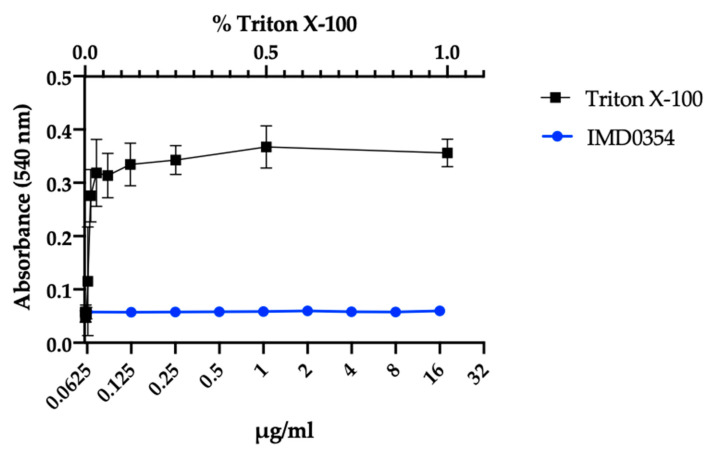
IMD0354 does not show hemolytic activity. Human red blood cells were incubated with serially diluted IMD0354 (0.0156–16μg/mL) and normalized to hemolysis of 1% Triton-X 100. Each sample was tested in triplicate (*n* = 3, ± S.D).

IMD0354 has been used in in vitro and in vivo studies as a potent NF-κB inhibitor and potential anti-cancer agent both dependent and independent of NF-κB inhibition [24,25,26,27]. We assessed the inhibitory activity of IMD0354 against the human liver hepatocellular carcinoma cell line (HepG2) and human kidney proximal tubular cell line (HKC-8) [39]. Consistent with previous studies, our results showed that IMD0354 has a median lethal concentration (LC_50_) of 1.1 µg/mL and 0.94 µg/mL, respectively. Noteworthy, IMD0354′s LC_50_ against cancer cells is approximately 16 times higher than its MIC to VRS1 (Figure 3). Additionally, it has been reported that IMD0354 does not induce detectable toxicity while showing significant efficacy in murine cancer models at concentrations as high as 30 mg/kg [26]. Consistently, IMD0354 was not toxic to *C. elegans* up to 2 µg/mL and showed 90% survival up to 64 µg/mL (Figure 4). Lastly, IMD0354 did not cause human red blood cell hemolysis up to 16 µg/mL (Figure 5). These results indicate that IMD0534 has a higher therapeutic index when used as an antimicrobial compared to toxicity.

### 2.4. IMD0354 Shows Bacterial Membrane Permeability at High Concentrations

**Figure 6:**
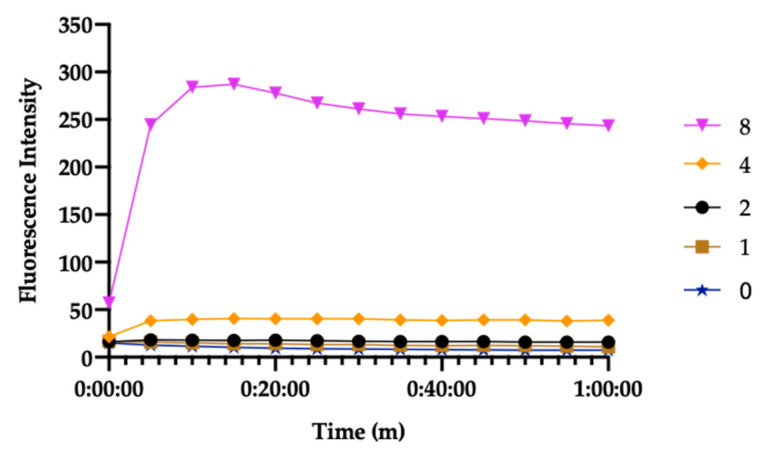
IMD0354 induces membrane permeabilization at high concentrations. VRS1 membrane permeabilization was measured spectrophotometrically by monitoring the uptake of SYTOX Green (excitation wavelength of 485 nm and an emission wavelength of 525 nm) during treatment with IMD0354 at various concentrations. The legend units are µg/mL. Each assay was tested in triplicate. (*n* = 3, ± S.D). Error bars are omitted for clarity.

Next, we tested whether IMD0534 affects bacterial membrane permeability using a membrane-impermeable DNA binding dye, SYTOX Green [40]. IMD0354 was not able to induce an increase in fluorescence up to 2 µg/mL. However, the VRS1 cells treated with equal or more than 4 µg/mL IMD0354 exhibited a rapid increase in SYTOX Green fluorescence, indicating that IMD0354 is able to permeabilize VRS1 membrane at high concentration (≥ 4 µg/mL). Together with the time-killing data (Figure 2), we conclude that, at high concentrations, IMD0354 kills bacteria by disrupting the bacterial membrane integrity (Figure 6). In contrast, at low concentrations, IMD0354 inhibits VRS1 growth rather than killing VRS1 and does not induce detectable membrane permeabilization. These results demonstrate that IMD0354 may have multiple antimicrobial mechanisms of action, possibly dependent on its concentration levels.

### 2.5. IMD0354 Inhibits Initial Cell Attachment in a Dose-Dependent Manner and Fully Inhibtis Biofilm Formation

**Figure 7:**
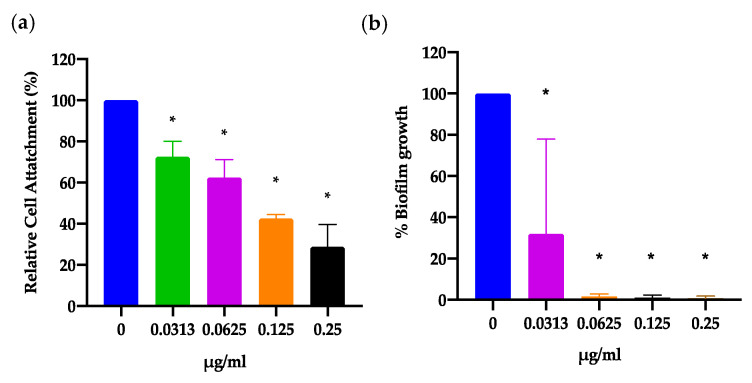
IMD0354 shows dose-dependent inhibition of initial cell attachment and complete inhibition of biofilm formation. (**a**) VRS1 cells were incubated in Brain Heart Infusion broth (BHI) 0.5% glucose at an optical density at 600 nm (OD_600_) of 1.0 for 1 h. Cells were washed three times with phosphate buffered saline (PBS) and then treated with XTT to measure cell attachment. Measurements were normalized to a non-treatment control (0 µg/mL). (**b**) VRS1 cells were incubated in BHI 0.5% glucose at an OD_600_ of 0.05 and indicated concentrations of IMD0354 for 24 h. At 24 h biofilm were washed 3 times with PBS. Total biofilm mass was measured using 1% crystal violet (*n* = 3, ± S.D.).

Biofilms are resistant to antibiotic treatment and are responsible for various chronic and recalcitrant infections [41]. Cell attachment is the initial stage of biofilm formation and development and is the main target in one of the principle strategies of biofilm management [42,43]. Therefore, it is clinically relevant to identify agents that inhibit biofilms during this critical step of initiation. Currently, there are already methods used to inhibit initial biofilm formation such as host-derived glycoproteinaceous film coating of medical implants and devices [44]. Likewise, molecular compounds such as aryl rhodanines or calcium chelators have also shown some success at inhibiting biofilm initial cell attachment [43]. To test the inhibitory activity of IMD0354 on biofilm initial cell attachment, we incubated a high density of VRSA strain VRS1 bacterial culture (~8 × 10^7^ CFU/mL) with various concentrations of IMD0354 (Figure 7) for 1 h and measured cell attachment using XTT (2–3-bis(2-methyloxy-4-nitro-5- sulfophenyl)-2H-tertazolium-5-carboxanilide) fluorescent dye. We found that IMD0354 is indeed able to block biofilm initial cell attachment in a dose-dependent manner with a greater than 60% inhibition at 4× MIC (0.25 µg/mL, (* *p* = 0.0059) (Figure 7a). Furthermore, we argue that the reduction in initial cell attachment is not due to antimicrobial activity given that we show that IMD0354 does not reduce cell viability after 1 h of incubation (Figure 2). To support these findings, we tested whether IMD0354 inhibits mature biofilm formation at concentrations which showed reduced initial cell attachment. As expected, we found that IMD0354 can completely inhibit biofilm formation beginning at a MIC concentration of 0.06 µg/mL (* *p* = 0.0005) with >50% inhibition at a sub-MIC concentration of 0.0313 µg/mL (* *p* = 0.0077). However, given that we showed that IMD0354 was unable to fully inhibit biofilm initial cell attachment but was still able to disrupt biofilm formation, we speculate that the inhibition of initial cell attachment is not the sole mechanism by which IMD0354 can hinder biofilm formation. Our working hypothesis is that the effect IMD0354 has on biofilm formation is due to the combined activity the compound has on bacterial growth and its ability to impede initial cell attachment. Furthermore, IMD0354 is unable to eradicate fully mature biofilm once established (data not shown).

### 2.6. IMD0354 Does Not Affect the Viability of Antibiotic-Tolerant Cells nor Synergize with Conventional Antibiotics

Membrane-active antimicrobial agents tend to show antimicrobial potency against non-growing dormant antibiotic-tolerant bacteria and synergism with other antibiotics [45]. Thus, we assessed if IMD0354 is potent against the antibiotic-tolerant *S. aureus* VRS1 and acts synergistically with conventional antibiotics. In this series of experiments, we isolated antibiotic-tolerant cells as previously described [46] and treated them with various concentrations of IMD0354 for 4 h. IMD0354 was not able to kill antibiotic-tolerant cells even at high concentrations such as 4 µg/mL (64× MIC) (Supplementary Figure S1). Next, we determined whether IMD0354 worked synergistically with other antibiotics against *S. aureus* VRS1. We performed various checkerboard assays, testing vancomycin, gentamicin, ciprofloxacin, and daptomycin in conjunction with IMD0354. Unlike in the case of Gram-negative bacteria [29], we did not find any synergy or antagonism between any antibiotics tested, only additive or indifference effects (data not shown).

## 3. Discussion

Here, we report the novel finding that the kinase inhibitor IMD0354 is able to prolong the life of *C. elegans* during a lethal MRSA infection [19]. We show that IMD0354 inhibits MRSA growth at MIC levels as low as 0.06 µg/mL and can inhibit growth of other multidrug-resistant Gram-positive bacteria including VRSA, VISA, and VRE. At high concentrations, IMD0354 permeabilizes Gram-positive bacterial membranes at concentrations ≥ 4 µg/mL (Figure 6).

In the previous report by Barker et al., IMD0354 was found to have no antimicrobial activity on its own against Gram-negative bacteria, which is consistent with our antimicrobial susceptibility test on Gram-negative ESKAPE pathogens (Table 1). Barker et al. demonstrated that the enhanced potency of colistin by IMD0354 results from its ability to reverse the colistin-resistance modification of lipid A of colistin-resistant bacteria [29]. Nonetheless, at this time, we cannot find any parallels between the Gram-negative mode of action of IMD0354 given that Gram-positive bacteria do not produce lipid A [47]. This may explain why we did not find any synergistic effect even with cell wall- or cell membrane-targeting antimicrobial agents, such as vancomycin or daptomycin, against Gram-positive VRS1.

In addition, IMD0354 inhibited the initial cell attachment for biofilm formation in a dose-dependent manner and completely inhibited biofilm formation at sub-MIC levels and above (Figure 7). It is possible that this phenotype is partially dependent on the antimicrobial activity of IMD0354. However, we find this unlikely given our experimental design and supporting data. For example, based on our killing kinetics assay, we show that IMD0354 has no antibacterial effect at 1 h post drug incubation. In addition, initial cell attachment assays are run using 10^2^ more bacteria than our killing kinetics studies. Therefore, we conclude that the reduction in initial cell attachment is independent of the antimicrobial activity by IMD0354. Biofilms are a significant threat given their high resistance to antibiotic therapy and common target of medical devices [41,42]. Biofilm formation and development consists of five stages, the first being cell attachment [42]. Attachment is controlled via various cell wall-anchoring proteins, including microbial surface components recognizing adhesive matrix molecules (MSCRAMMs) [42]. Our working hypothesis is that IMD0354 targets these associated genes or proteins and thus results in a decrease in biofilm attachment.

Furthermore, we also found that IMD0354 is a more potent antimicrobial than a cell toxicity agent. Previous studies have shown that IMD0354 exhibits anti-cancer activity by inhibiting cell invasion, viability, as well as acting as an adjuvant with other chemotherapy drugs [25,26,27]. In particular, our studies show that the average LC_50_ of IMD0354 toward two cancerous cell lines is 1.04 µg/mL, approximately 17× greater than its MIC of 0.06 µg/mL. IMD0354 is known to selectively suppress the proliferation of cancerous cells over normal cells [48]. For instance, unlike on neoplastic mast cells, it did not affect the proliferation of normal human mast cells at 1 µM (0.4 µg/mL) [48]. Furthermore, various groups have used IMD0354 in murine in vivo models from 1 mg/kg up to 30 mg/kg over several weeks and have reported no detectable toxicity [24,26]. These previous in vitro and in vivo results demonstrate that IMD0354 is relatively non-toxic to normal cells. Consistently, we observed the cytotoxic effect of IMD0354 on cancerous cells at 1 µg/mL (Figure 3), while it did not cause cytotoxicity to the model animal *C. elegans* at ~7 µg/mL (Figure 1b). It is worth noting the MIC of IMD0354 against the VRSA strain VRS1 is 0.06 µg/mL (Table 2), which is about one order of magnitude lower than its effective concentration on cancer cell lines. Given these findings, we propose that IMD0354 has greater promise in terms of being repurposed as an antimicrobial rather than an anti-cancer agent.

The antibacterial activity of other anti-cancer drugs, such as mitomycin C and cisplatin, has been validated and these drugs have been proposed as antimicrobial candidates against multidrug-resistant bacteria [49,50]. For instance, mitomycin C demonstrates an LC_50_ of 27 µM (9.03 µg/mL) against HepG2 cancer cells and a MIC ranging between 0.2–15 µg/mL to multiple Gram-negative and Gram-positive bacteria [49]. Alternatively, cisplatin shows an IC_50_ of 2 µg/mL against HepG2 cancer cells and a MIC > 50 µg/mL for both Gram-positive and Gram-negative bacteria [50,51]. Nonetheless, low-dose administration of cisplatin to septic mice improves their bacterial clearance [52]. From these studies, we find that certain anti-cancer agents might have antimicrobial activity at concentrations similar to or greater than their anti-cancer activity. In contrast, IMD0354 has an LC_50_ of 1.1 µg/mL to HepG2 cells and a MIC of 0.06 µg/mL. These data demonstrate that IMD0354 has a greater antimicrobial to anti-cancer activity ratio than both mitomycin C and cisplatin.

Interestingly, the antimicrobial and anti-cancer mechanism of action (MOA) of mitomycin C and cisplatin appear to be similar as they both cross-link mammalian and bacterial cell DNA, thus leading to cell death [49,50]. On the other hand, the anti-cancer MOA of IMD0354 has been shown to be both NF-κB dependent and independent [24,25,26,27]. Given that bacteria have no NF-κB it is reasonable to assume that the antibacterial MOA of IMD0354 is different from its anti-cancer activity. Importantly, this information allows us to speculate that IMD0354 could be a promising lead compound that can be structurally optimized to abate or nullify anti-cancer activity while retaining its antimicrobial properties.

In addition to anti-cancer activity, IMD0354 has other bioactivities. Onai et al. and Sugita et al. used IMD0354 to directly inhibit NF-κB and subsequently target inflammation [24,28]. From these in vitro studies, we glean that IMD0354 can significantly inhibit cytokine production at 1 µM (0.4 µg/mL) [24], nearly six times more than IMD0354′s MIC (0.06 µg/mL). Furthermore, in vivo data from Onai et al. showed a significant reduction in inflammation in a rat myocardial ischemia/reperfusion injury model after treatment with IMD0354. In these studies rats, were treated with either 1 mg/kg, 5 mg/kg, or 10 mg/kg of IMD0354 over 4 weeks. After treatment, it was found that only 5 mg/kg and 10 mg/kg had significant differences in reducing infarction size. We therefore suggest that there is a low concentration window in which even IMD0354 can be administered as an antibiotic with low cross-activation of other bioactivities.

Overall, the continued evolution of antibiotic resistance to last-resort therapeutics such as vancomycin persist as a primary threat. The *C. elegans*–MRSA high-throughput screening (HTS) system has become an invaluable tool in drug discovery research as this model is unique in its ability to find antimicrobial kinase inhibitors that would otherwise be neglected due to their toxicity, such as NF-κB inhibitors. Moving forward, we find it important to distinguish the structural relationship between NF-κB inhibition and antimicrobial effect. Further research into analogs would be beneficial in advancing our understanding of the mechanism of action of kinase inhibitors, which can illuminate new antimicrobial targets against multidrug-resistant bacteria. In additional, experiments testing the efficacy of IMD0354 as an antimicrobial in a murine in vivo model would be insightful. Given IMD0354′s low MIC, we speculate that there may be a tritiated dose that does not induce significant NF-κB inhibition but is still able to inhibit bacterial growth. However, at this time, these experiments fall outside the focus of our study.

## 4. Materials and Methods

### 4.1. Bacterial Strains and Growth Conditions

All strains used for these studies are listed in Table 4. All VISA and VRSA strains were grown overnight in trypsin soy broth (TSB) at 37 °C, followed by shaking at 180–225rpm. All *Enterococcal* strains were grown overnight in Brain Heart Infusion broth (BHI) at 37 °C, followed by shaking at 180–225 rpm.

### 4.2. Drugs and Antibiotics

IMD0354 (Tocris 2611), gentamicin, daptomycin, and vancomycin (Sigma Aldrich) stocks were dissolved to 10 mg/mL in DMSO. Ciprofloxacin (Sigma Aldrich) was dissolved to 10 mg/mL in 0.1 N HCl.

### 4.3. Minimum Inhibitory Concentration Assay

Minimum Inhibitory Concentration assays were carried out as described by the Clinical and Laboratory Standard Institute [53]. In brief, bacterial strains grown overnight in appropriate media for 20–23 h were diluted to 1 × 10^6^ CFU/mL in Mueller–Hinton Broth (MHB, BD Difco, pH: 7.3 ± 0.1). In a 96-well plate 50 µL of diluted culture was added to 50 µL of serial two-fold diluted drug in MHB to a final concentration of 5 × 10^5^ CFU/mL. All assays were performed in triplicate. Experimental plates were incubated for 20–22 h at 37 °C. Optical density at 600 nm (OD_600_) was measured using a spectrophotometer (SpectraMax M2, Molecular Devices) as a measure of bacterial growth. MIC was defined as OD_600_ ≤ 0.1 after background subtraction.

### 4.4. C. elegans Infection Assay for Compound Screening

All compounds were screened as previously described in Kim et al. 2014 [19]. In brief, *glp-4*(bn2); *sek-1*(km4) worm embryos were synchronized by plating 2000 L1 worms on SK agar plates with HB101 bacteria as a food source at 15 °C for four days until they reached gravid adult stage. Eggs were harvested and hatched in M9 buffer at 15 °C for 48 h. L1 stage worms were then transferred onto SKHB101 plates and incubated at 25 °C for 52 h to induce sterility. Sterile young adult stage worms were harvested using M9 buffer and sorted into black, clear-bottom, 384-well plates (Corning no. 3712) containing compounds at 15 worms/well using Copa Biosort Instrument. *S. aureus* MW2 bacteria was grown overnight in TSB at 37 °C with agitation. A static culture was inoculated by seeding 100 µL of an overnight culture in 10 mL of fresh TSB, sealed to produce anaerobic conditions, and incubated at 37 °C overnight without agitation. We have found that MW2 grown anaerobically elicits a greater infection mortality rate in *C. elegans.* Furthermore, anaerobically grown MRSA MW2 cells express different virulence gene patterns [54]. Static MW2 was added to *C. elegans*-compound 384-well plates at a final concentration of OD_600_ 0.04. Final well composition consisted of 70% M9 buffer, 19% Sheath solution (Union Biometrica Part no. 300-5101-000), 10% TSB, and 1% DMSO or compounds dissolved in DMSO. After a 5-day incubation at 25 °C worms were washed using a multiplate washer and incubated overnight at 37 °C with Sytox Orange dissolved in M9 at a final concentration of 0.7 µM. The following day, all plates were imaged using an Image Xpress Micro automated microscope (Molecular Devices), capturing both transmitted light and TRITC (535 nm excitation, 610 nm emission) fluorescent images using a 2× objective.

### 4.5. C. elegans Dose-Dependent Toxicity Assay

In a black, clear-bottom, 96-well plate (Corning, no. 3690) IMD0354 was serially diluted to a final volume of 50 µL using M9. N2 worms were sterilized by growing to young adult stage fed on RNAi *cdc 25.1* activated by 1 mM of IPTG [55] for 48 h at 25 °C. *cdc25.1* is an integral part of germ cell mitosis. Mutations of *cdc25.1* inhibits germ line cell division producing *C. elegans* incapable of laying eggs [56]. Young adult worms were washed 3 times with 50 mL of M9 and diluted to an average of 21 worms ± 7 /25 µL using a multichannel pipette. An additional 25 µL of heat killed OP50 were added to each well to a make a final volume of 100 µL and OD_600_ of 0.5. All assays were performed in duplicate. Experimental plates were incubated for 24 h at 25 °C. Worms were then washed using a 405 LS microplate washer (BioTek) and incubated with 0.7 µM SYTOX Orange for an additional 24 h at 25 °C. Each plate was then imaged using an Xpress Micro automated microscope (Molecular Devices) capturing both transmitted light and TRITC (535 nm excitation, 610 nm emission) fluorescent images using a 2X objective. Surviving worms were considered those with no TRITC signal relative to the control.

### 4.6. Bacterial Time-Course Killing Assay

Strain VRS1 [32], grown overnight in TSB medium for 20–23 h, was diluted 1:1000 (1 × 10^6^ CFU/mL) in MHB (BD Difco, pH 7.3 ± 0.1) in a deep 96-well plate. 250 µL of diluted culture was added to 250 µL of serial two-fold diluted drug in MHB to a final concentration of 5 × 10^5^ CFU/mL. At time 0, 1 h, 2 h, 3 h, 4 h, and 24 h, 50-μL samples were removed, serially diluted by 10-fold steps, and spot-plated on MHB agar (BD Difco) plates to enumerate the number CFU/mL. These experiments were conducted in triplicate. Experimental plates were incubated for 20–22 h at 37 °C.

### 4.7. Human Blood Hemolysis

Hemolytic activity of IMD0354 on human erythrocytes was evaluated using a previously described method with modifications [57]. 10% human erythrocytes were purchased from Rockland Immunochemicals (Limerick, PA, USA). The erythrocytes were diluted to 4% with phosphate buffered saline (PBS), and 50 μL was added to 50 μL of two-fold serial dilutions of compounds in PBS, 0.2% DMSO (negative control), or 1% Triton-X 100 (positive control) in a 96-well plate. The plate was incubated at room temperature for 1 h and then centrifuged at 500× *g* for 5 min. 50 μL of the supernatant was transferred to a fresh 96-well plate and absorbance of supernatants was measured at 540 nm. Percent hemolysis was calculated using the following equation: (A540nm of compound treated sample—A540nm of 0.1% DMSO treated sample)/(A540nm of 1% Triton X-100 treated sample—A540nm of 0.1% DMSO treated sample) × 100. These experiments were conducted in triplicate.

### 4.8. Antibiotic-Tolerant Cell Killing

*S. aureus* VRS1 [32] antibiotic-tolerant cells were acquired by growing liquid cultures >18 h to stationary-phase at 37 °C in 25 mL of TSB [46]. Stationary-phase VRS1 cell tolerance to various antibiotics was previously proven by Kim et al. 2018 [46]. In brief, overnight cultures were washed three times with PBS (pH: 7.4) and diluted to a final concentration of 1.0 × 10^6^ CFU/mL. 500 µL of washed cells were added to 500 µL of indicated concentrations of drug in 2 mL deep-dish 96-well plates and incubated at 37 °C with agitation for 1, 2, 3, and 4 h. In order to wash any residual drug from sample time points, 400 µL of sample was collected every hour and centrifuged at 15,000 rpm for 3 min and suspended with 400 µL of fresh PBS. 100 µL of washed samples were serially diluted and spot-plated on MHB agar plates to measure antibiotic-tolerant cell CFU/mL. These experiments were conducted in triplicate.

### 4.9. SYTOX Green Membrane Permeability Assay

These studies were conducted as previously described in Kim et al. 2018 [19]. In brief, black, clear-bottom, 96-well plates (Corning no. 3904, Corning, NY, USA) were filled with 50 μL of PBS (pH: 7.4) containing 2× the indicated concentration of antibiotics. Stationary-phase VRS1 cells prepared as described in the antibiotic-tolerant cell killing assay were washed 3 times with equal volumes of PBS. Washed cells were then adjusted to OD_600_ 0.4 (~2 × 10^7^ CFU/mL) with PBS. SYTOX Green (Molecular Probes, Waltham, MA, USA) was added to 10 mL of the diluted bacterial suspension to a final concentration of 5 μM and incubated for 30 min at room temperature in the dark. 50 μL of the bacteria/SYTOX Green mixture was added to each well of the 96-well plates containing antibiotics. Fluorescence was measured at room temperature using a spectrophotometer (SpectraMax M2, Molecular Devices, Sunnyvale, CA, USA), with excitation and emission wavelengths of 485 nm and 525 nm, respectively. All experiments were conducted in triplicate.

### 4.10. Biofilm Initial Cell Attachment Assay

*S. aureus* strainVRS1 [32] was grown overnight (> 18 h) in BHI (Sigma, pH: 7.4 ± 0.2). Absorbance at OD_600_ was measured and adjusted to 0.2 in BHI + 0.1% glucose medium. 100 µL of bacteria was added to 2× drug concentrations being tested producing a final volume of 200 µL, OD_600_ of 0.1 (~ 8 × 10^7^ CFU/mL) and 1× drug concentration. The plates were then incubated at 37 °C for 1 h. Media were then pipetted out and the wells were washed 3 times with PBS to remove any non-adherent planktonic cells. Biofilm initial cell attachment was measured as described in Biswajit et al. 2016 [58]. In brief, colorimetric quantification of the inhibition of biofilm initial cell attachment was done using XTT [2–3-bis(2-methyloxy-4-nitro-5- sulfophenyl)-2H-tertazolium-5-carboxanilide] assay kit following manufacture instructions with minor adjustments (Sigma-Aldrich, MO, USA). 180 µL of fresh TSB and 20 µL of XTT solution were added to each well and the plates were again incubated for 2 h at 37 °C. Absorbance at 450 nm was measured and each experimental well was normalized to a non-treatment control. Each biological replicate was done in quadruplicates. One replicate was done in octuplet.

### 4.11. Biofilm Inhibition Assay

*S. aureus* VRS1 [32] was grown overnight (>18 h) in BHI (Sigma, pH: 7.4 ± 0.2). Absorbance at OD_600_ was measured and adjusted to 0.1 in BHI + 0.1% glucose medium. 100 µL of bacteria was added to 2× drug concentrations being tested producing a final volume of 200 µL, OD_600_ of 0.05 (~4 × 10^7^ CFU/mL) and 1× drug concentration. The plates were then incubated at 37 °C for 24 h. Media were then pipetted out and the wells were washed 3 times with PBS to remove any non-adherent planktonic cells. Biofilm inhibition was measured using crystal violet (CV) [59]. In brief, plates were incubated with 1% CV for 15 min at room temperature. Plates were then washed 3 times with PBS and dissolved with 200 µL of 30% acetic acid. Absorbance at 550 nm was measured and each experimental well was normalized to a non-treatment control. Each biological replicate was done in octuplet.

### 4.12. Mammalian Cancer Cell Viability Assay

Two-fold concentration drug plates were prepared using Dulbecco’s Modified Eagle’s medium (DMEM) with 10% FBS. HepG2 or HKC-8 cells were grown in DMEM 10% FBS to confluency and seeded onto 96-well drug plates at 1.0 × 10^6^ cells/mL and 0.4 × 10^6^ cells/mL, respectively. Drug and cell plate were then incubated for 22 h at 37 °C and 5% CO_2_. At 22 h, 10 µL of WST-1 (Roche, Sigma) was added to each well, following manufacturer’s directions, and incubated for an additional 2 h. Plate absorbance was read at 450 nm. Samples were normalized to a non-treatment control. All experiments were conducted in triplicate.

## 5. Conclusions

In conclusion, we report that, at low concentrations (≤ 2 µg/mL), IMD0354 can act both as a bactericidal and bacteriostatic against VISA, VRSA, and VRE strains, while, at high concentrations (≥8 µg/mL), IMD0354 demonstrates bactericidal activity. IMD0354 does not show any hemolytic activity at concentrations up to 16 µg/mL and shows no toxicity to *C. elegans* up to 2 µg/mL and 90% survival at > 64µ/mL. Our data reveal that the antimicrobial mechanism of IMD0354 at high concentrations ≥ 4 µg/mL is membrane permeabilization. However, we are still unclear what the MOA is at low concentrations. Importantly, we find that IMD0354 is a more potent antimicrobial than anti-cancer agent. Moving forward, we believe that the further development of this compound is important. Nonetheless, further research to develop this multi-bioactive compound will require distinguishing the structural relationship between NF-κB inhibition, anti-cancer, and antimicrobial effects in order to overcome toxicity and cross-reaction side effects.

## Figures and Tables

**Table 1:** Minimum inhibitory concentration (μg/mL) of IMD0354 to selected ESKAPE pathogens.

Strain	MIC
*Pseudomonas aeruginosa* PA14	>64
*Klebsiella pneumoniae* WGLW2	>64
*Acinetobacter baumannii* ATCC 17978	16
*Enterobacter aerogenes* EAE 2625	>64
*Enterococcus faecalis* MMH 594	0.25
*Enterococcus faecium* E007	0.125

**Table 2:** Minimum inhibitory concentration (μg/mL) of NF-κB inhibitors IMD0354 against vancomycin-resistant *Staphylococcus aureus* (VRSA) and vancomycin-intermediate *Staphylococcus aureus* (VISA) clinical strains.

	IMD0354		Vancomycin	
Strain	MIC	MBC	MIC	MBC
VRS1	0.06	2	>64	>64
VISA 215	0.25	0.5	8	8
VISA 216	0.06	2	8	16
VISA 217	0.125	0.5	8	8
VISA 218	0.06	0.5	8	8
VISA 219	0.06	1	8	16
VISA 220	0.25	2.0	8	16
VISA 221	0.06	1.0	8	8
VISA 222	0.125	2.0	8	8
VISA 223	0.125	1.0	4	8
VISA 224	0.25	2	4	16
VISA 225	0.125	0.25	4	8
VISA 226	0.06	0.125	4	4
VISA 227	0.25	0.5	8	8
VISA 228	0.125	0.5	4	8

**Table 3:** Minimum inhibitory concentration (μg/mL) of NF-κB inhibitors IMD0354 against vancomycin-resistant enterococci (VRE) clinical strains.

	IMD0354		Vancomycin	
Strain	MIC	MBC	MIC	MBC
EM C68 [35]	0.25	8	64	>64
EM D366 [36]	0.25	8	64	>64
EM WB312 [37]	0.25	8	64	>64
EM WC176 [37]	0.25	8	64	>64
EL V583 [38]	0.25	8	64	>64

EM: Enterococcus faecium, EL: Enterococcus faecalis.

**Table 4:** Bacterial strains used in this study.

Bacterial Name	Strain
vancomycin-intermediate *Staphylococcus aureus*	VRS1 [32]
vancomycin-intermediate *Staphylococcus aureus* (clinical isolates)	VISA 215, 216, 217, 218, 219, 220, 221, 222, 223, 224, 225, 226, 227, 228
vancomycin-resistant *Enterococcus faecium* (clinical isolates)	C68 [35], D366 [36], WB312 [37], WC176 [37]
vancomycin-resistant *Enterococcus faecalis* (clinical isolate)	V583 [38]
methicillin-resistant *Staphylococcus aureus*	MW2
*Klebsiella pneumoniae*	WGLW2
*Acinetobacter baumannii*	ATCC 17978
*Enterococcus faecalis*	MMH 594
*Enterococcus faecium*	E007
*Pseudomonas aeruginosa*	PA14

## Supplementary Materials

The following are available online at https://www.mdpi.com/2079-6382/9/10/665/s1, Figure S1: IMD0354 does not kill VRS1 antibiotic-tolerant cells.

## References

[1] David M.Z., Daum R.S. (2010). Community-Associated Methicillin-Resistant *Staphylococcus aureus:* Epidemiology and Clinical Consequences of an Emerging Epidemic. Clin. Microbiol. Rev..

[2] Lowy F.D. (1998). *Staphylococcus aureus* infections. N. Engl. J. Med..

[3] Lakhundi S., Zhang K. (2018). Methicillin-Resistant *Staphylococcus aureus:* Molecular Characterization, Evolution, and Epidemiology. Clin. Microbiol. Rev..

[4] Stogios P.J., Savchenko A. (2020). Molecular mechanisms of vancomycin resistance. Protein Sci..

[5] Hiramatsu K., Aritaka N., Hanaki H., Kawasaki S., Hosoda Y., Hori S., Fukuchi Y., Kobayashi I. (1997). Dissemination in Japanese hospitals of strains of *Staphylococcus aureus* heterogeneously resistant to vancomycin. Lancet.

[6] Howe R.A., Bowker K.E., Walsh T.R., Feest T.G., MacGowan A.P. (1998). Vancomycin-resistant *Staphylococcus aureus*. Lancet.

[7] Hidayat L.K., Hsu D.I., Quist R., Shriner K.A., Wong-Beringer A. (2138). High-dose vancomycin therapy for methicillin-resistant *Staphylococcus aureus* infections: Efficacy and toxicity. Arch. Intern. Med..

[8] Leclercq R., Derlot E., Duval J., Courvalin P. (1988). Plasmid-mediated resistance to vancomycin and teicoplanin in *Enterococcus faecium*. N. Engl. J. Med..

[9] Uttley A.H., Collins C.H., Naidoo J., George R.C. (1988). Vancomycin-resistant enterococci. Lancet.

[10] Dougherty T.J., Pucci M.J. (2011). Antibiotic Discovery and Development.

[11] Munita J.M., Arias C.A. (2016). Mechanisms of Antibiotic Resistance. Microbiol. Spectr..

[12] Arthur M., Molinas C., Courvalin P. (1992). The VanS-VanR two-component regulatory system controls synthesis of depsipeptide peptidoglycan precursors in *Enterococcus faecium* BM4147. J. Bacteriol..

[13] Meziane-Cherif D., Saul F.A., Haouz A., Courvalin P. (2012). Structural and functional characterization of VanG D-Ala:D-Ser ligase associated with vancomycin resistance in *Enterococcus faecalis*. J. Biol. Chem..

[14] Tattevin P., Arvieux C., Michelet C. (2007). Alternative agents for the treatment of invasive infections due to methicillin-resistant *Staphylococcus aureus* strains with reduced susceptibility to vancomycin. Arch. Intern. Med..

[15] Gomes D.M., Ward K.E., LaPlante K.L. (2015). Clinical implications of vancomycin heteroresistant and intermediately susceptible *Staphylococcus aureus*. Pharmacotherapy.

[16] Rybak M.J., Hershberger E., Moldovan T., Grucz R.G. (2000). In vitro activities of daptomycin, vancomycin, linezolid, and quinupristin-dalfopristin against *Staphylococci* and Enterococci, including vancomycin- intermediate and -resistant strains. Antimicrob. Agents Chemother..

[17] Chen C.-J., Huang Y.-C., Shie S.-S. (2020). Evolution of Multi-Resistance to Vancomycin, Daptomycin, and Linezolid in Methicillin-Resistant *Staphylococcus aureus* Causing Persistent Bacteremia. Front. Microbiol..

[18] Nannini E., Murray B.E., Arias C.A. (2010). Resistance or decreased susceptibility to glycopeptides, daptomycin, and linezolid in methicillin-resistant *Staphylococcus aureus*. Curr. Opin. Pharmacol..

[19] Kim W., Zhu W., Hendricks G.L., Van Tyne D., Steele A.D., Keohane C.E., Fricke N., Conery A.L., Shen S., Pan W. (2018). A new class of synthetic retinoid antibiotics effective against bacterial persisters. Nature.

[20] Kim S.M., Escorbar I., Lee K., Fuchs B.B., Mylonakis E., Kim W. (2020). Anti-MRSA agent discovery using *Caenorhabditis elegans*-based high-throughput screening. J. Microbiol..

[21] Kim W., Steele A.D., Zhu W., Csatary E.E., Fricke N., Dekarske M.M., Jayamani E., Pan W., Kwon B., Sinitsa I.F. (2018). Discovery and Optimization of nTZDpa as an Antibiotic Effective against Bacterial Persisters. ACS Infect. Dis..

[22] Kim W., Zou G., Hari T.P.A., Wilt I.K., Zhu W., Galle N., Faizi H.A., Hendricks G.L., Tori K., Pan W. (2019). A selective membrane-targeting repurposed antibiotic with activity against persistent methicillin-resistant *Staphylococcus aureus*. Proc. Natl. Acad. Sci. USA.

[23] Kim W., Zou G., Pan W., Fricke N., Faizi H.A., Kim S.M., Khader R., Li S., Lee K., Escorba L. (2020). The Neutrally Charged Diarylurea Compound PQ401 Kills Antibiotic-Resistant and Antibiotic-Tolerant *Staphylococcus aureus*. mBio.

[24] Onai Y., Suzuki J.-I., Kakuta T., Maejima Y., Haraguchi G., Fukasawa H., Muto S., Itai A., Isobe M. (2004). Author NotesInhibition of IkappaB phosphorylation in cardiomyocytes attenuates myocardial ischemia/reperfusion injury. Cardiovasc. Res..

[25] Kim S., Ko D., Lee Y., Jang S., Lee Y., Lee I.Y., Kim S. (2019). Anti-cancer activity of the novel 2-hydroxydiarylamide derivatives IMD-0354 and KRT1853 through suppression of cancer cell invasion, proliferation, and survival mediated by TMPRSS4. Sci. Rep..

[26] Kinose Y., Sawada K., Makino H., Tomonori O., Tomoko M., Noriko S., Tomoyuki F., Eiichi M., Koji N., Ikuko S. (2015). IKKβ Regulates VEGF Expression and Is a Potential Therapeutic Target for Ovarian Cancer as an Antiangiogenic Treatment. Mol. Cancer Ther..

[27] Gomez-Cabrero A., Wrasidlo W., Reisfeld R.A. (2013). IMD-0354 targets breast cancer stem cells: A novel approach for an adjuvant to chemotherapy to prevent multidrug resistance in a murine model. PLoS ONE.

[28] Sugita A., Ogawa H., Azuma M., Muto S., Honjo A., Yanagawa H., Nishioka Y., Tani K., Itai A., Sone S. (2009). Antiallergic and anti-inflammatory effects of a novel I kappaB kinase beta inhibitor, IMD-0354, in a mouse model of allergic inflammation. Int. Arch. Allergy Immunol..

[29] Barker W.T., Nemeth A.M., Brackett S.M., Basak A.K., Chandler C.E., Jania L.A., Zuercher W.J., Melander R.J., Koller B.H., Ernst R.K. (2019). Repurposing Eukaryotic Kinase Inhibitors as Colistin Adjuvants in Gram-Negative Bacteria. ACS Infect. Dis..

[30] Rice L.B. (2008). Federal funding for the study of antimicrobial resistance in nosocomial pathogens: No ESKAPE. J. Infect. Dis..

[31] Pendleton J.N., Gorman S.P., Gilmore B.F. (2013). Clinical relevance of the ESKAPE pathogens. Expert Rev. Anti-Infect. Ther..

[32] Weigel L.M., Clewell D.B., Gill S.R., Clark N.C., McDougal L.K., Flannagan S.E., Kolonay J.F., Shetty J., Killgore G.E., Tenover F.C. (2003). Genetic analysis of a high-level vancomycin-resistant isolate of *Staphylococcus aureus*. Science.

[33] Centers for Disease Control and Prevention (2019). CDC & FDA Antibiotic Resistance (AR) Isolate Bank.

[34] Gupta V., Singla N., Behl P., Sahoo T., Chander J. (2015). Antimicrobial susceptibility pattern of vancomycin resistant enterococci to newer antimicrobial agents. Indian J. Med. Res..

[35] Carias L.L., Rudin S.D., Donskey C.J., Rice L.B. (1998). Genetic linkage and cotransfer of a novel, vanB-containing transposon Tn5382) and a low-affinity penicillin-binding protein 5 gene in a clinical vancomycin-resistant *Enterococcus faecium* isolate. J. Bacteriol..

[36] Williamson R., Al-Obeid S., Shlaes J.H., Goldstein F.W., Shlaes D.M. (1989). Inducible resistance to vancomycin in *Enterococcus faecium* D366. J. Infect. Dis..

[37] Thorisdottir A.S., Carias L.L., Marshall S.H., Green M., Zervos M.J., Giorgio C., Mermel L.A., Boyce J.M., Medeiros A.A., Fraimowet H. (1994). IS6770, an enterococcal insertion-like sequence useful for determining the clonal relationship of clinical *enterococcal* isolates. J. Infect. Dis..

[38] Evers S., Sahm D.F., Courvalin P. (1993). The vanB gene of vancomycin-resistant *Enterococcus faecalis* V583 is structurally related to genes encoding D-Ala:D-Ala ligases and glycopeptide-resistance proteins VanA and VanC. Gene.

[39] Racusen L.C., Monteil C., Sgrignoli A., Lucskay M., Marouillat S., Rhim J.G., Morin J.P. (1997). Cell lines with extended in vitro growth potential from human renal proximal tubule: Characterization, response to inducers, and comparison with established cell lines. J. Lab. Clin. Med..

[40] Kim W., Conery A.L., Rajamuthiah R., Fuchs B.B., Ausubel F.M., Mylonakis E. (2015). Identification of an antimicrobial agent effective against methicillin-resistant *Staphylococcus aureus* persisters using a fluorescence-based screening strategy. PLoS ONE.

[41] Percival S.L., Suleman L., Vuotto C., Donelli G. (2015). Healthcare-associated infections, medical devices and biofilms: Risk, tolerance and control. J. Med. Microbiol..

[42] Moormeier D.E., Bayles K.W. (2017). *Staphylococcus aureus* biofilm: A complex developmental organism. Mol. Microbiol..

[43] Chung P.Y., Toh Y.S. (2014). Anti-biofilm agents: Recent breakthrough against multi-drug resistant *Staphylococcus aureus*. Pathog. Dis..

[44] Bjarnsholt T., Ciofu O., Molin S., Givskov M., Høiby N. (2013). Applying insights from biofilm biology to drug development—Can a new approach be developed?. Nat. Rev. Drug Discov..

[45] Hurdle J.G., O’Neill A.J., Chopra I., Lee R.E. (2010). Targeting bacterial membrane function: An underexploited mechanism for treating persistent infections. Nat. Rev. Microbiol..

[46] Kim W., Fricke N., Conery A.L., Fuchs B.B., Rajamuthiah R., Jayamani E., Vlahovska P.M., Ausubel F.M., Mylonakis E. (2016). NH125 kills methicillin-resistant *Staphylococcus aureus* persisters by lipid bilayer disruption. Future Med. Chem..

[47] Raetz C.R.H., Whitfield C. (2002). Lipopolysaccharide endotoxins. Annu. Rev. Biochem..

[48] Tanaka A., Konno M., Muto S., Kambe N., Morii E., Nakahata T., Itai A., Matsuda H. (2005). A novel NF-kappaB inhibitor, IMD-0354, suppresses neoplastic proliferation of human mast cells with constitutively activated c-kit receptors. Blood.

[49] Kwan B.W., Chowdhury N., Wood T.K. (2015). Combatting bacterial infections by killing persister cells with mitomycin C. Environ. Microbiol..

[50] Chowdhury N., Wood T.L., Martinez-Vazquez M., García-Contreras R., Wood T.K. (2016). DNA-crosslinker cisplatin eradicates bacterial persister cells. Biotechnol. Bioeng..

[51] Yang E.B., Tang W.Y., Zhang K., Cheng L.Y., Mack P.O. (1997). Norcantharidin inhibits growth of human HepG2 cell-transplanted tumor in nude mice and prolongs host survival. Cancer Lett..

[52] Li Y., Wang Z., Ma X., Shao B., Gao X., Zhang B., Xu G., Wei Y. (2014). Low-dose cisplatin administration to septic mice improves bacterial clearance and programs peritoneal macrophage polarization to M1 phenotype. Pathog. Dis..

[53] CLSI (2012). M07-A9: Methods for Dilution Antimicrobial Susceptibility Tests for Bacteria That Grow Aerobically.

[54] Fuchs S., Pané-Farré J., Kohler C., Hecker M., Engelmann S. (2007). Anaerobic gene expression in *Staphylococcus aureus*. J. Bacteriol..

[55] Yuen G.J., Ausubel F.M. (2018). Both live and dead Enterococci activate *Caenorhabditis elegans* host defense via immune and stress pathways. Virulence.

[56] Kim J., Lee A.-R., Kawasaki I., Strome S., Shim Y.-H. (2009). A mutation of *cdc-25.1* causes defects in germ cells but not in somatic tissues in *C. elegans*. Mol. Cells.

[57] Rajamuthiah R., Jayamani E., Conery A.L., Fuchs B.B., Kim W., Johnston T., Vilcinskas A., Ausubel F.M., Mylonakis E. (2015). A Defensin from the Model Beetle *Tribolium castaneum* Acts Synergistically with Telavancin and Daptomycin against Multidrug Resistant *Staphylococcus aureus*. PLoS ONE.

[58] Mishra B., Golla R.M., Lau K., Lushnikova T., Wang G. (2016). Anti-Staphylococcal Biofilm Effects of Human Cathelicidin Peptides. ACS Med. Chem. Lett..

[59] O’Toole G.A. (2011). Microtiter dish biofilm formation assay. J. Vis. Exp..

