# Supplementary material for: New Antimicrobial Bioactivity against Multidrug-Resistant Gram-Positive Bacteria of Kinase Inhibitor IMD0354
Source: Antibiotics (Basel). 2020 Oct 1;9(10):665. doi: 10.3390/antibiotics9100665 (PMC7601562; doi:10.3390/antibiotics9100665)
Supplement: Supplementary file 1 [file antibiotics-09-00665-s001.zip › antibiotics-934335-supplementary.docx]

**Supplementary Figures**

**Supplementary Figure 1.** IMD0354 does not kill VRS1 antibiotic-tolerant cells. An overnight culture of VRS1 was washed twice in PBS. After washing isolated VRS1 antibiotic-tolerant cells where incubated with 1, 2, or 4µg/ml of IMD0354 for 4 h. Bithionol at 32µg/ml was used as a positive control. At 4 h each sample was additionally washed to remove any excess compound and spot plated on MHB agar plates to enumerate total antibiotic-tolerant cells remaining. Each assay was performed in triplicate. (n=3, ± S.D)
